# Serum and follicular anti-Mullerian hormone levels in women with polycystic ovary syndrome (PCOS) under metformin

**DOI:** 10.1186/1757-2215-3-16

**Published:** 2010-07-21

**Authors:** Angela Falbo, Morena Rocca, Tiziana Russo, Antonietta D'Ettore, Achille Tolino, Fulvio Zullo, Francesco Orio, Stefano Palomba

**Affiliations:** 1 Department of Obstetrics & Gynecology, University "Magna Graecia" of Catanzaro, Catanzaro, Italy; 2 Department of Obstetrics & Gynecology, University "Federico II" of Naples, Naples, Italy; 3 Endocrinology, "Parthenope" University, Naples, Italy

## Abstract

**Background:**

No data regarding metformin effects on follicular fluid anti-Müllerian hormone (AMH) levels were to date available in literature. The aim of the present study was to evaluate in patients with polycystic ovary syndrome (PCOS) whether metformin administration affects serum and follicular AMH levels, and whether this is related to ovarian response to the treatment.

**Methods:**

Twenty young patients with PCOS who had received metformin were enrolled. Ten patients were anovulatory (Met-anov group), whereas the other 10 were ovulatory (Met-ov group) but had failed to conceive. Further untreated PCOS (PCOS controls, n. 10) and healthy controls (non-PCOS controls, n. 10) who were scheduled for laparoscopic surgery were enrolled. In each subjects, clinical and biochemical evaluations were performed. AMH concentrations in blood and antral follicular fluid were assayed.

**Results:**

In both Met-anov and Met-ov groups, and without difference between them, serum androgens and AMH, and indices of insulin resistance were significantly (*p *< 0.05) improved after treatment. On the other hand, significant differences (*p *< 0.05) between the two groups were detected with respect to the same biochemical parameters in antral follicular fluid. In the Met-anov group, no significant correlation was observed between AMH concentrations in the follicular fluid and variation in serum androgens, AMH and insulin resistance indexes; whereas in Met-ov group significant correlations were detected between AMH levels in the follicular fluid and variation in serum androgens, AMH and insulin resistance indexes.

**Conclusions:**

Metformin administration in patients with PCOS exerts a differential action on the ovarian AMH levels on the basis of ovulatory response. Changes in AMH levels in antral follicular fluid during metformin treatment could be involved in the local mechanisms mediating the ovulatory restoration.

## Background

Anti-Müllerian hormone (AMH) is a member of the transforming growth factor-β (TGF-β) family. In females, AMH is mainly secreted by the granulosa cells of ovarian early developing follicles [1].

The expression of AMH is localized in granulosa cells of primary, pre-antral and small antral follicles, suggesting an important role of AMH in human folliculogenesis [2]. Since AMH is secreted exclusively in the gonads, its serum concentrations in females are thought to reflect the size of the ovarian follicle pool [2,3].

Polycystic ovary syndrome (PCOS), one of the most common endocrine disorders in women of childbearing age [4-6], is characterized by a marked increase in pre-antral follicles number [7]. To date, controversial data are available regarding the relationship between the high serum AMH levels and the pre-antral follicles number in PCOS patients [8-12]. Thus, is still unknown if the AMH excess in PCOS is secondary to the increase in pre-antral follicles number, or if an intrinsic increased AMH production by the granulosa cells is the cause of follicular arrest in PCOS.

A direct correlation between ovarian antral follicle counts and ovarian volume with hyperinsulinemia was referred in PCOS women [13,14]. Furthermore, it is unclear if the PCOS-related hyperinsulinemic state could induce the development of antral follicles by increasing the sensitivity of granulosa cells to FSH determining an higher number of follicles and a major ovarian volume [15-17].

Metformin, an insulin-sensitizing drugs recently introduced for the treatment of women with PCOS, has been demonstrated to induce regular menstrual cycles and to increase ovulation in patients with PCOS, although the efficacy of the drug is extremely variable both between different PCOS populations and within the same population [18].

A recent experimental study was conducted with the aim to evaluate whether the efficacy of metformin in patients with PCOS is related to a systemic hormonal-metabolic improvement or to a local action on the ovary [19]. The authors found that, irrespectively to systemic effects, the efficacy of metformin in inducing ovulation in patients with PCOS was probably due to a direct action of the drug on a "sensitive" ovary.

At the moment, the few studies aimed to assess the effects of metformin administration in PCOS patients on serum AMH levels reported controversial findings [9,20-22], and any data is actually available in literature regarding the metformin effects on follicular fluid AMH levels. Based on these considerations, the aim of the present study was to evaluate in patients with PCOS whether metformin administration affects serum and follicular AMH levels, and whether this effect is related to ovarian response to the treatment.

## Methods

The study was approved by the Institutional Review Board of the Department of Obstetrics and Gynecology, University "Magna Graecia" of Catanzaro, Italy. The purpose of the protocol was explained carefully to all the patients and written consent was obtained before the study began.

Twenty young normal weight patients with PCOS who had received metformin treatment to induce ovulation and, then, scheduled for laparoscopy were enrolled at our Academic Centre of Reproductive Medicine and Surgery between October 2001 and February 2010, and studied as cases. The majority of the subjects had participated in our earlier studies [19,23].

All patients with PCOS had received the same metformin regimen (two 850 mg tablets daily) for one year. On the basis of the response to treatment received, cases were distinguished according to ovarian response to metfomin into two groups (Met-anov and Met-ov groups). Specifically, Met-anov group (n. 10) was composed of PCOS patients who remained anovulatory despite treatment, and Met-ov group (n. 10) included PCOS women who resulted normally cycled under metformin treatment (for at least six cycles) but had failed to conceive.

According to our Institutional guidelines, subjects from the Met-anov group were scheduled for ovarian drilling procedure, whereas subjects from the Met-ov group were scheduled for diagnostic laparoscopy in order to exclude potential infertility/subfertility factors.

Other 20 patients were enrolled as controls. Of them, 10 were untreated patients with PCOS [24,25], affected by uterine fibroids and scheduled for laparoscopic myomectomy (PCOS controls), whereas other 10 normally cycled women were scheduled for diagnostic laparoscopy because they referred chronic pelvic pain (non-PCOS controls).

In PCOS patients, PCOS diagnosis was based initially on the presence of both chronic anovulation and clinical and/or biochemical hyperandrogenism [25], even if all patients with PCOS originally had bilateral polycystic ovaries (PCO) [24]. In healthy controls, ovulatory cycles were confirmed by biochemistry, and clinical and/or biochemical hyperandrogenism and PCO were systematically excluded.

Were considered exclusion criteria for all subjects: an age less than 18 or greater than 35 years; a body mass index (BMI, kg/m^2^) less than 18 or greater than 25; major medical disorders and/or current or previous use of hormonal and/or metabolic drugs; tubal or male factor infertility or sub-fertility investigated with hysterosalpingography and standard semen analysis, respectively (Male Infertility Best Practice Policy Committee of the American Urological Association, 2006; Practice Committee of the American Society for Reproductive Medicine 2006); any organic pelvic diseases at laparoscopy or diseases potentially affecting the ovarian environment and/or function (including endometriosis, leiomyomas, and so on); and the intention to start a diet or a specific programme of physical activity. In addition, subjects with dominant follicle(s) (follicles with a diameter equal or higher than 10 mm) and/or with persistent corpora lutea and/or functional cysts at transvaginal ultrasound performed before surgery were excluded. Clinical, biochemical, and ultrasonographic parameters at baseline or before metformin administration were acquired retrospectively, whereas all other data were evaluated prospectively at the hospital admission.

Clinical evaluation, blood sampling, transvaginal ultrasonography, and laparoscopy were performed in each subject. Clinical evaluation consisted of gynecological examination, anthropometric measurements and Ferriman-Gallwey score calculation. Biochemical assessment consisted of complete hormonal, including evaluation of serum follicle stimulating hormone (FSH), luteinizing hormone (LH), thyroid-stimulating hormone (TSH), prolactin (PRL), estradiol (E_2_), P, 17-OH-progesterone (17-OHP), total testosterone (T), androstenedione (A), dehydroepiandrosterone sulfate (DHEAS), and sex-hormone binding globulin (SHBG)], and metabolic evaluation, including evaluation of fasting glucose and insulin levels. Insulin resistance was evaluated using the homeostasis model analysis (HOMA) [fasting glucose (mmol/L) × fasting insulin (μU/mL)/22.5] and the fasting glucose-to-insulin ratio (GIR, mg/10^-4^U). The free androgen index (FAI) [T (nmol/l)/SHBG × 100] was also calculated for each participant.

Serum and follicular fluid AMH levels were assessed by using a second generation enzyme immunoassay (AMH-EIA kit; Immunotech A Beckman Coulter Company, Marseilles, France), according to the supplier's instructions. The intra-assay and inter-assay coefficients of variation (CV) for each biochemical or hormonal parameter were evaluated, and the values of the CVs ranged from 1.2 to 5.8%.

Finally, the ovarian dimensions, volume and morphology and the number of antral follicles (follicular diameter ranged from 2 to 9 mm) were evaluated bilaterally by transvaginal ultrasonography. The antral follicle number per ovary, defined as the average for the total number of antral follicles counted from both ovaries, was also calculated.

All laparoscopic interventions were performed by the same experienced operator (F.Z.) during the early follicular phase for ovulatory subjects and randomly in anovulatory patients. Firstly, the antral follicles on the ovarian surface were visualized and each one was aspirated with a 1 mL syringe and a 26 gauge needle. Follicular fluid of antral follicles was collected from both ovaries in each patient, it was transferred to the laboratory on dry ice, and purified from the granulosa cells. Thereafter, the remaining follicular fluid was centrifuged, and the supernatant was stored at -20°C until it underwent biochemical analysis.

As scheduled, ovarian diathermy and myomectomy were performed in Met-anov and PCOS control group, respectively.

### Statistical analysis

Continuous variables were tested for normality using the Kolmogrov-Smirnov test resulting normally distributed and were expressed as the mean ± standard deviation (SD).

Data were analyzed with one-way analysis of variance (ANOVA) and ANOVA for repeated measures, and the Bonferroni test was used for post-hoc analysis.

For categorical variables, the Pearson chi-square test was performed; Fisher's exact test was used for the frequency tables when more than 20% of the expected values were lower than five.

A simple linear regression analysis was used to establish the relationships between the AMH in the follicular fluid, and the variation (Δ) in plasma T levels (ΔT), HOMA (ΔHOMA), and AMH (ΔAMH). A bivariate two-tailed correlation analysis was performed by calculating the Spearman's coefficient (Spearman's rho, r), and the significance of the correlation was set at the 0.05 level.

The level of statistical significance was set at *p *< 0.05 for all statistical analyses. The Statistics Package for Social Sciences (SPSS 14.0.1, 18 Nov 2005; SPSS Inc., Chicago, IL) was used for all calculations.

## Results

The criteria of the National Institutes of Health (NIH), the criteria of the European Society for Human Reproduction (ESHRE)/American Society of Reproductive Medicine (ASRM) [5] and those of the Androgen Excess & PCOS Society (AEPS) [26] were all satisfied in our sample.

The clinical, hormonal, and metabolic data from all groups at baseline and their variation after treatment are shown in Table 1.

**Table 1:** Clinical, hormonal, and metabolic data in Met-anov and Met-ov groups, and in PCOS and non-PCOS controls.

Group	Met-anov		Met-ov		PCOS controls	Non-PCOS controls
	***Before treatment***	***Δ***	***Before treatment***	***Δ***		

**Age (years)**	27.40 ± 3.21	0.12 ± 0.15	28.08 ± 3.45	0.08 ± 0.29	27.83 ± 3.61	28.17 ± 3.58
**BMI (kg/m^2^)**	23.00 ± 1.58	-0.11 ± 0.25	22.97 ± 1.37	-0.06 ± 0.10	22.84 ± 1.64	23.09 ± 1.58
**WHR**	0.76 ± 0.12	0.01 ± 0.02	0.77 ± 0.10	-0.00 ± 0.06	0.76 ± 0.10	0.75 ± 0.98
**Ferriman-Gallwey score**	12.67 ± 2.70°	-1.84 ± 0.45	12.42 ± 2.43°	-0.09 ± 0.03	12.0 ± 2.98°	3.25 ± 1.91
**FSH (mIU/mL)**	5.85 ± 1.57	0.06 ± 0.03	5.86 ± 1.32	-0.03 ± 0.04	6.04 ± 1.39	5.50 ± 1.60
**LH (mIU/mL)**	11.89 ± 3.87°	-0.12 ± 0.31	12.56 ± 3.45°	-0.76 ± 0.25	12.56 ± 3.48°	10.43 ± 2.48
**TSH (μU/mL)**	2.94 ± 0.75	-0.02 ± 0.07	2.99 ± 0.72	-0.06 ± 0.05	2.81 ± 0.77	3.01 ± 0.65
**PRL (ng/mL)**	8.90 ± 2.13	0.08 ± 0.13	9.17 ± 1.83	1.26 ± 0.06	8.26 ± 2.13	9.20 ± 1.93
**E_2 _(pg/mL)**	52.45 ± 16.43	0.53 ± 18.60	49.79 ± 14.98	0.26 ± 0.08	50.18 ± 11.00	52.24 ± 8.65
**P (ng/mL)**	1.38 ± 0.43	-0.02 ± 0.04	1.33 ± 0.44	-0.11 ± 0.09	1.40 ± 0.42	1.42 ± 0.36
**17-OHP (μg/L)**	2.25 ± 0.50	-0.28 ± 0.07	2.06 ± 0.46	-0.13 ± 0.07	2.20 ± 0.47	1.85 ± 0.41
**T (ng/mL)**	4.65 ± 1.15°	-1.14 ± 0.19	4.62 ± 1.13°	-0.35 ± 0.12	4.71 ± 1.02°	1.10 ± 0.29
**A (ng/mL)**	4.82 ± 1.91°	-1.63 ± 0.08	4.40 ± 1.13°	-0.20 ± 0.08	4.80 ± 1.14°	1.84 ± 0.45
**DHEAS (ng/mL)**	2674.10 ± 189.7°	-9.8 ± 0.25	2703.43 ± 204.42°	-17.55 ± 0.11	2696.66 ± 215.77°	1792.50 ± 253.84
**SHBG (nmol/L)**	32.41 ± 3.35 °†	0.49 ± 0.32	30.41 ± 1.88°	0.75 ± 1.06	30.50 ± 2.20°	49.45 ± 5.96
**FAI (%)**	14.50 ± 4.13°	-4.24 ± 1.39	14.63 ± 4.06°	-4.57 ± 0.07	14.78 ± 5.20°	3.73 ± 1.44
**Fasting glucose (mmol/L)**	4.60 ± 0.49	-0.35 ± 0.06	4.61 ± 0.47	-0.08 ± 0.08	4.52 ± 0.50	4.65 ± 0.46
**Fasting insulin (μU/mL)**	16.20 ± 4.82°†	-0.53 ± 0.22	16.51 ± 3.57°	-2.31 ± 0.11	16.30 ± 4.39°	14.36 ± 2.07
**GIR (mg/10^-4^U)**	5.64 ± 1.18°†	0.36 ± 0.10	5.40 ± 1.59°	1.17 ± 0.14	5.21 ± 1.41°	7.45 ± 1.24
**HOMA**	3.20 ± 0.61°†	-0.04 ± 0.17	3.73 ± 0.61°	-0.41 ± 0.11	3.17 ± 0.69°	2.90 ± 0.71
**AMH (ng/mL)**	5.23 ± 1.59°†	-2.0 ± 1.05	5.75 ± 1.59°	-7.41 ± 2.32	3.92 ± 1.62°	1.56 ± 1.02

*p < 0.05 *vs*. before treatment assessment; †*p *< 0.05 *vs*. Met-ov group; ^*p *< 0.05 *vs*. PCOS controls; °*p *< 0.05 *vs*. non-PCOS controls.

In both Met-anov and Met-ov groups, levels of T, A, SHBG, and fasting insulin, as well as FAI, GIR, HOMA, and AMH were improved significantly (*p <*0.05) after treatment.

A significant difference (*p *< 0.05) between Met-anov and Met-ov groups was observed at baseline and after metformin with regards to the serum levels of SHBG, fasting insulin, GIR, HOMA, and AMH before treatment (Table 1).

In both Met-anov and Met-ov groups, serum levels of LH, T, A, DHEAS, SHBG, fasting insulin, and AMH as well as the Ferriman-Gallwey score, FAI, GIR and HOMA, were significantly (p < 0.05) better than those in PCOS controls and significantly (*p *< 0.05) worse than those in non-PCOS controls (Table 1).

No difference in the mean variation of any clinical, hormonal or metabolic parameter was observed between Met-anov and Met-ov groups (Table 1).

**Figure 1:**
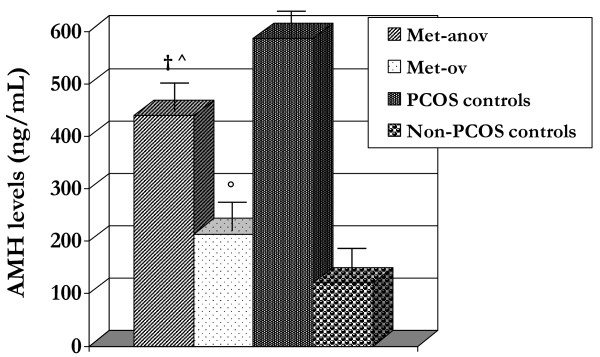
**AMH levels in the follicular fluid of the antral follicles**. †*p *< 0.05 *vs*. Met-ov group; ^*p *< 0.05 *vs*. PCOS controls; °*p *< 0.05 *vs*. non-PCOS controls.

In Figure 1 are shown the AMH concentrations in the follicular fluid of the antral follicles. Significant differences (*p *< 0.05) were observed between Met-anov and Met-ov groups in AMH levels in the antral follicular fluid (Figure 1). Moreover, AMH levels in the antral follicles were significantly different (p < 0.05) for both Met-anov group vs. PCOS controls and Met-ov group vs. non-PCOS controls (Figure 1).

The correlations between AMH levels in the follicular fluid and ΔT, ΔHOMA and ΔAMH, in Met-anov and Met-ov groups are shown in Table 2.

**Table 2:** Linear correlation between AMH concentrations in the follicular fluid, and variation (Δ) in serum T, HOMA and AMH, in the Met-anov and Met-ov groups.

	Met-anov group (n = 10)		Met-ov group (n = 10)	
	***r***	***p***	***r***	***p***

**Follicular fluid AMH**				
**ΔT**	-0.543	0.213	-0.701	0.039
**ΔHOMA**	0.121	0.185	0.645	0.044
**ΔAMH**	-0.543	0.315	-0.821	0.026

No significant correlation was observed between AMH concentrations in the follicular fluid, and ΔT, ΔHOMA and ΔAMH, in the Met-anov group. On the contrary, significant correlations were detected between AMH levels in the follicular fluid and ΔT (*r *= -0.701; *p *= 0.039), ΔHOMA (*r *= 0.645; *p *= 0.044), and ΔAMH (*r *= -0.821; *p *= 0.026).

## Discussion

The present experimental study firstly evaluated the effect of metformin administration on AMH concentrations assayed both on serum and follicular fluid in women affected by PCOS. Our data confirmed [27,28] that AMH levels were significantly higher in PCOS patients than in healthy controls. A plausible hypothesis for this figure is that the increased AMH levels in PCOS are the results of the increased number of small ovarian follicles [29,30]. In this regard, a direct and a significant correlation between follicle number and serum AMH levels has been demonstrated by some authors [8-10,12], even if the hypotheses provided for this correlation were not univocal [27,28].

Interesting results were obtained by the evaluation of AMH levels in PCOS women who were treated with metformin. In particular, we used as study model PCOS patients who had a different response to metformin administration in order to clarify the role of AMH in the ovarian response to the treatment.

As already reported [1], our data seem to suggest that AMH might play a key role in the intra-ovarian mechanisms regulating the ovarian function. In fact, significant changes in serum AMH levels in PCOS patients ovulating under metformin, such as in those remaining anovulatory despite treatment were detected. The reason for the reduction in AMH concentrations after metformin remains still controversial.

In a prospective study [20], metformin acutely improved insulin resistance indexes and restored ovarian morphology, whereas no effect of the metformin-induced improved insulin-sensitivity and AMH levels was observed. These data [20] are strongly limited by the very small sample size and the short-term observation period. Moreover, Piltonen *et al. *[9], in a prospective study, showed that the AMH levels, the number of antral follicles and the ovarian volume were reduced after metformin administration. In addition, a positive correlation was found between serum AMH levels and both follicle count and androgen levels [9]. These correlations were successively confirmed [12], and a further relationship between AMH levels and insulin resistance indexes was demonstrated in untreated PCOS patients. On the other hand, in a recent prospective, randomized, double-blind 26 week long-term study [21], AMH levels in untreated PCOS women seemed to be associated positively with testosterone, and negatively with DHEAS and C-peptide levels. Moreover, the same authors showed that 6 months of androgen suppression by either metformin or low-dose dexamethasone treatment failed to influence circulating AMH levels [21].

The current study, confirming and extending our previous data [31], suggests that metformin acts on ovarian AMH levels with additive and direct mechanism of action. In fact, the effects of metformin at ovarian site did not reflect those observed at systemic levels. Significant difference in intraovarian AMH levels was observed within PCOS patients who received metformin on the basis of clinical response, even if women ovulating under metformin maintained higher follicular AMH levels than healthy controls. Thus, it is possible to hypothesize that metformin exerts a peripheral effect on the ovary by lowering AMH concentration that is detrimental for clinical response to the treatment. On the other hand, a slight effect on follicular AMH level was also observed in unresponsive PCOS patients, in fact significant difference in AMH levels was observed between anovulatory PCOS women who had received metformin and untreated anovulatory PCOS patients.

A simple linear regression analysis was performed to establish the relationship between AMH in the follicular fluid and the systemic response to the treatment, which included ΔT and ΔHOMA as indicators for improved hyperandrogenism and insulin resistance, respectively, and serum ΔAMH.

As already shown [32], ovaries in our population with PCOS seemed to have a differential sensitivity to metformin, and that an improved biochemical response to metformin by a "sensitive" ovary could be decisive for the clinical response mediated by AMH. In this regard, ovulatory patients with PCOS had significant correlations between the AMH levels in follicular fluid and the variation in plasma T and AMH levels and the variation in HOMA, respectively. On the contrary, patients with PCOS who were anovulatory under metformin seemed to have a local "resistance" to the treatment, and no significant correlation between the variation in any systemic factors and follicular AMH levels was observed in these patients.

## Conclusions

Metformin administration in anovulatory patients with PCOS exerts a differential action on the ovarian AMH levels on the basis of ovulatory response. Changes in AMH levels in antral follicular fluid during metformin treatment could be involved in the local mechanisms mediating the ovulatory restoration. Further well designed studies on a larger sample are needed before obtaining definitive conclusions.

## Competing interests

The authors declare that they have no competing interests.

## Authors' contributions

SP conceived of the study, and participated in its design and coordination. FA conceived of the study, participated in the study design and performed the statistical analysis. MR, TR and AD participated in the patients' enrolment. FO, AT and FZ participated in the manuscript drafting and critical discussion. All authors read and approved the final manuscript.

## References

[B1] La Marca A Broekmans FJ Volpe A Fauser BC Macklon NS ESHRE Special Interest Group for Reproductive Endocrinology--AMH Round Table Anti-Mullerian hormone (AMH): what do we still need to know? Hum Reprod 2009 24 2264 2275 10.1093/humrep/dep210 19520713

[B2] Weenen C Laven JS Von Bergh AR Cranfield M Groome NP Visser JA Kramer P Fauser BC Themmen AP Anti-Müllerian hormone expression pattern in the human ovary: potential implications for initial and cyclic follicle recruitment Mol Hum Reprod 2004 10 77 83 10.1093/molehr/gah015 14742691

[B3] Van Rooij IA Broekmans FJ te Velde ER Fauser BC Bancsi LF de Jong FH Themmen AP Serum anti-Müllerian hormone levels: a novel measure of ovarian reserve Hum Reprod 2002 17 3065 3071 10.1093/humrep/17.12.3065 12456604

[B4] Ehrmann DA Polycystic ovary syndrome N Engl J Med 2005 352 1223 1236 10.1056/NEJMra041536 15788499

[B5] Rotterdam ESHRE/ASRM-Sponsored PCOS Consensus Workshop Group Revised 2003 consensus on diagnostic criteria and long-term health risks related to polycystic ovary syndrome Fertil Steril 2004 81 19 25 10.1016/j.fertnstert.2003.10.004 14711538

[B6] Balen AH Laven JS Tan SL Dewailly D Ultrasound assessment of the polycystic ovary: international consensus definitions Hum Reprod Update 2003 9 505 514 10.1093/humupd/dmg044 14714587

[B7] Franks S Stark J Hardy K Follicle dynamics and anovulation in polycystic ovary syndrome Hum Reprod Update 2008 14 367 378 10.1093/humupd/dmn015 18499708

[B8] Laven JS Mulders AG Visser JA Themmen AP De Jong FH Fauser BC Anti-Müllerian hormone serum concentrations in normoovulatory and anovulatory women of reproductive age J Clin Endocrinol Metab 2004 89 318 323 10.1210/jc.2003-030932 14715867

[B9] Piltonen T Morin-Papunen L Koivunen R Perheentupa A Ruokonen A Tapanainen JS Serum anti-Müllerian hormone levels remain high until late reproductive age and decrease during metformin therapy in women with polycystic ovary syndrome Hum Reprod 2005 20 1820 1826 10.1093/humrep/deh850 15802325

[B10] Catteau-Jonard S Pigny P Reyss AC Decanter C Poncelet E Dewailly D Changes in serum anti-mullerian hormone level during low-dose recombinant follicular-stimulating hormone therapy for anovulation in polycystic ovary syndrome J Clin Endocrinol Metab 2007 92 4138 4143 10.1210/jc.2007-0868 17698904

[B11] Catteau-Jonard S Jamin SP Leclerc A Gonzalès J Dewailly D di Clemente N Anti-Mullerian hormone, its receptor, FSH receptor, and androgen receptor genes are overexpressed by granulosa cells from stimulated follicles in women with polycystic ovary syndrome J Clin Endocrinol Metab 2008 93 4456 4461 10.1210/jc.2008-1231 18697861

[B12] Chen MJ Yang WS Chen CL Wu MY Yang YS Ho HN The relationship between anti-Mullerian hormone, androgen and insulin resistance on the number of antral follicles in women with polycystic ovary syndrome Hum Reprod 2008 23 952 957 10.1093/humrep/den015 18256110

[B13] Pache TD de Jong FH Hop WC Fauser BC Association between ovarian changes assessed by transvaginal sonography and clinical and endocrine signs of the polycystic ovary syndrome Fertil Steril 1993 59 544 549 8458455 10.1016/s0015-0282(16)55797-5

[B14] Carmina E Orio F Palomba S Longo RA Lombardi G Lobo RA Ovarian size and blood flow in women with polycystic ovary syndrome and their correlations with endocrine parameters Fertil Steril 2005 84 413 419 10.1016/j.fertnstert.2004.12.061 16084883

[B15] Loucks TL Talbott EO McHugh KP Keelan M Berga SL Guzick DS Do polycystic-appearing ovaries affect the risk of cardiovascular disease among women with polycystic ovary syndrome? Fertil Steril 2000 74 547 552 10.1016/S0015-0282(00)00695-6 10973653

[B16] Legro RS Chiu P Kunselman AR Bentley CM Dodson WC Dunaif A Polycystic ovaries are common in women with hyperandrogenic chronic anovulation but do not predict metabolic or reproductive phenotype J Clin Endocrinol Metab 2005 90 2571 2579 10.1210/jc.2004-0219 15713728

[B17] Fulghesu AM Villa P Pavone V Guido M Apa R Caruso A Lanzone A Rossodivita A Mancuso S The impact of insulin secretion on the ovarian response to exogenous gonadotropins in polycystic ovary syndrome J Clin Endocrinol Metab 1997 82 644 648 10.1210/jc.82.2.644 9024269

[B18] Palomba S Falbo A Zullo F Orio F Evidence-based and potential benefits of metformin in the polycystic ovary syndrome: a comprehensive review Endocr Rev 2009 30 1 50 10.1210/er.2008-0030 19056992

[B19] Palomba S Falbo A Russo T Orio F Tolino A Zullo F Systemic and local effects of metformin administration in patients with polycystic ovary syndrome (PCOS): relationship to the ovulatory response Hum Reprod 2010 25 1005 1013 10.1093/humrep/dep466 20106839

[B20] Bayrak A Terbell H Urwitz-Lane R Mor E Stanczyk FZ Paulson RJ Acute effects of metformin therapy include improvement of insulin resistance and ovarian morphology Fertil Steril 2007 87 870 875 10.1016/j.fertnstert.2006.08.096 17224152

[B21] Carlsen SM Vanky E Fleming R Anti-Müllerian hormone concentrations in androgen-suppressed women with polycystic ovary syndrome Hum Reprod 2009 24 1732 1738 10.1093/humrep/dep074 19342396

[B22] Fábregues F Castelo-Branco C Carmona F Guimerá M Casamitjana R Balasch J The effect of different hormone therapies on anti-müllerian hormone serum levels in anovulatory women of reproductive age Gynecol Endocrinol 2010 in press 10.3109/09513590.2010.487595 20500102

[B23] Palomba S Falbo A Battista L Russo T Venturella R Tolino A Orio F Zullo F Laparoscopic ovarian diathermy vs clomiphene citrate plus metformin as second-line strategy for infertile anovulatory patients with polycystic ovary syndrome: a randomized controlled trial Am J Obstet Gynecol 2010 202 e1 8 10.1016/j.ajog.2009.11.042 20096821

[B24] Adams J Polson DW Franks S Prevalence of polycystic ovaries in women with anovulation and idiopathic hirsutism Br Med J 1986 293 355 359 10.1136/bmj.293.6543.355 PMC1341046 3089520

[B25] Zawadzki JK Dunaif A Dunaif A, Givens JR, Haseltine FP, Merriam GR Diagnostic criteria for polycystic ovary syndrome: towards a rational approach Polycystic ovary syndrome 1992 Boston: Blackwell 337 384

[B26] Azziz R Carmina E Dewailly D Diamanti-Kandarakis E Escobar-Morreale HF Futterweit W Janssen OE Legro RS Norman RJ Taylor AE Witchel SF Androgen Excess Society Positions statement: criteria for defining polycystic ovary syndrome as a predominantly hyperandrogenic syndrome an Androgen Excess Society guideline J Clin Endocrinol Metab 2006 91 4237 4245 10.1210/jc.2006-0178 16940456

[B27] Pellatt L Rice S Mason HD Anti-Mullerian hormone and polycystic ovary syndrome: a mountain too high? Reproduction 2010 139 825 833 10.1530/REP-09-0415 20207725

[B28] Pellatt L Hanna L Brincat M Galea R Brain H Whitehead S Mason H Granulosa cell production of anti-Müllerian hormone is increased in polycystic ovaries J Clin Endocrinol Metab 2007 92 240 245 10.1210/jc.2006-1582 17062765

[B29] Das M Gillott DJ Saridogan E Djahanbakhch O Anti-Mullerian hormone is increased in follicular fluid from unstimulated ovaries in women with polycystic ovary syndrome Hum Reprod 2008 23 2122 2126 10.1093/humrep/den185 18550512

[B30] Pigny P Jonard S Robert Y Dewailly D Serum anti-Mullerian hormone as a surrogate for antral follicle count for definition of the polycystic ovary syndrome J Clin Endocrinol Metab 2006 91 941 945 10.1210/jc.2005-2076 16368745

[B31] Falbo A Orio F Venturella R Rania E Materazzo C Tolino A Zullo F Palomba S Does metformin affect ovarian morphology in patients with polycystic ovary syndrome? A retrospective cross-sectional preliminary analysis J Ovarian Res 2009 2 5 10.1186/1757-2215-2-5 19480717 PMC2694802

[B32] Palomba S Falbo A Russo T Orio F Tolino A Zullo F Systemic and local effects of metformin administration in patients with polycystic ovary syndrome (PCOS): relationship to the ovulatory response Hum Reprod 2010 25 1005 1013 10.1093/humrep/dep466 20106839

